# Development of a highly sensitive Gaussia luciferase immunoprecipitation assay for the detection of antibodies against African swine fever virus

**DOI:** 10.3389/fcimb.2022.988355

**Published:** 2022-09-14

**Authors:** Jingjing Ding, Jifei Yang, Daoyuan Jiang, Yanyang Zhou, Chenxi Li, Yanhua Li

**Affiliations:** ^1^ College of Veterinary Medicine, Yangzhou University, Yangzhou, China; ^2^ African Swine Fever Regional Laboratory of China (Lanzhou), State Key Laboratory of Veterinary Etiological Biology, Lanzhou Veterinary Research Institute, Chinese Academy of Agricultural Sciences, Lanzhou, China; ^3^ Comparative Medicine Research Institute, Yangzhou University, Yangzhou, China; ^4^ Jiangsu Co-Innovation Center for Prevention and Control of Important Animal Infectious Diseases and Zoonoses, Yangzhou, China

**Keywords:** African swine fever virus, luciferase immunoprecipitation system, gaussia luciferase, p30, p54, Serological diagnosis

## Abstract

In recent years, African swine fever (ASF) has caused a devastating blow to the swine industry globally. Since no effective vaccine is available, strict biosafety measures and rapid diagnosis are the most effective strategies for ASF control. ASFV p30 is one of the most antigenic viral proteins that have been widely used in the field for serological diagnosis of ASF infection. In this study, we developed a luciferase immunoprecipitation system (LIPS) assay for the detection of ASFV antibodies in pig serum using Gaussia luciferase (GLuc)-tagged p30 as a diagnostic antigen. The optimal GLuc-p30 input of 10^7^ luminance units (LU) and optimal serum dilution factor of 1/100 were set to achieve the highest P/N ratio. Based on 87 ASFV-positive and negative pig sera, the cutoff value of the S/N ratio could be set between 2.298 and 30.59 to achieve 100% sensitivity and 100% specificity. Moreover, the diagnostic sensitivity of this LIPS is comparable to that of a commercial enzyme-linked immunosorbent assay (ELISA) and the specificity of LIPS is even superior to the tested ELISA. In conclusion, we have established a LIPS assay for ASFV antibody detection, which could be a potential method for ASFV diagnosis in laboratories and farms.

## Introduction

African swine fever (ASF) is an acute, hemorrhagic, and highly contagious swine disease caused by the African swine fever virus (ASFV) which is the only arthropod-borne DNA virus in the *Asfarviridae* family. It was first diagnosed in Kenya in 1909 and reported as a swine disease with clinical symptoms indistinguishable from those of classical swine fever (CSF) in 1921 (Cwynar et al., 2019). On August 3, 2018, ASF emerged in the Liaoning province of China and gradually spread in this largest pig-producing and pork-consuming country in the world (Zhou et al., 2018). It is listed as one of the notifiable diseases by the World Organization for Animal Health (OIE) and is also recognized as a class I animal epidemic disease in China (Blome et al., 2020). The ASFV genome is about 170-190 kb in length and encodes more than 150 open reading frames (ORFs). ASFV can be classified according to their virulence into high pathogenicity, medium virulence, low virulence, and asymptomatic infection strains. The highly pathogenic viral infection caused almost 100% morbidity and mortality (Wang et al., 2021). Due to its high morbidity and mortality, ASF causes devastating losses to the swine industry and has important socio-economic significance.

Lacking effective vaccines and antiviral treatments, ASF control attaches great importance to efficient diagnosis methods of ASFV (Wu et al., 2020). ASFV-specific antibodies appear soon after infection and can persist in convalescent animals for months to years (Gallardo et al., 2019). Recently, the low virulent strains of genotype II ASFV and genotype I ASFV have been identified in domestic swine herds in China (Sun et al., 2021; Sun et al., 2021). In comparison with the prevalent high virulent strains, the low virulent variants cause mild and delayed clinical symptoms but can shed viruses *via* the oral and rectal routes (Sun et al., 2021). Diagnosis methods targeting viral antigens or DNA do not always guarantee the identification of infected animals with chronic or inapparent forms of the disease. Under this circumstance, the serological diagnosis would be more informative and important (Kazakova et al., 2017). The OIE recommended serological diagnostic methods for ASFV include indirect fluorescent antibody (IFA) test, indirect enzyme-linked immunosorbent assays (ELISA), immunoblotting test, or immunoperoxidase staining (https://www.woah.org/fileadmin/Home/eng/Health_standards/tahm/3.08.01_ASF.pdf). Although IFA shows great sensitivity, it is only suitable to be used as a confirmatory test due to its complicated procedure and time-consuming (Wu et al., 2020). Many commercialized ELISA tests have been widely used for ASFV serological diagnosis, but their sensitivity is not comparable to IFA. Currently, it is urgent to develop rapid, high-throughput, cost-effective, and easy-to-implement serological methods.

To date, several ASFV proteins have been explored as diagnostic targets for the development of ELISA assays, including p72, p54, p30, CD2v, and p35 (Perez-Filgueira et al., 2006; Gimenez-Lirola et al., 2016; Lv et al., 2021; Cao et al., 2021; Shi et al., 2021; Yu et al., 2021; Yuan et al., 2021; Zhao et al., 2022; Wang et al., 2022; Carmina et al., 2022). ASFV p30 is a phosphorylated structural protein encoded by the early gene *CP204L*. It is distributed in the inner membrane of viral particles with a size of 22.4 kDa (Afonso et al., 1992). Serological studies of ASFV by radioimmunoprecipitation showed that the virus-specific antibodies detected in sera during the early stage of viral infection (3 to 6 days after infection) were mainly specific to the p30 (Kazakova et al., 2017). Furthermore, p30 demonstrates a high degree of antigenic conservation among virus strains in different geographic locations, and it induced the production of antibodies in all naturally infected and experimentally infected pigs (Oviedo et al., 1997). Given these characteristics, p30 is an ideal diagnostic target for the early detection of ASFV infection. ASFV p54 encoded by the gene *E183L* was identified as a highly antigenic protein during infection (Cubillos et al., 2013). So, p54 is another attractive candidate for ASF detection.

The luciferase immunoprecipitation system (LIPS) is a liquid phase immunoassay that uses a luciferase-tagged antigen to capture antigen-specific antibodies (Burbelo et al., 2005). The luciferase-tagged antigens used for LIPS assay are usually produced in mammalian cells, allowing them to go through necessary post-translational modifications as well as proper folding and exposure of the antigenic epitopes. Due to the low natural background of bioluminescence, soluble crude cell lysates of the luciferase-tagged recombinant proteins can be extracted from transfected cells and used without purification (Haljasmagi et al., 2020). As a liquid-phase immunoassay, the antigens in their native conformation enable LIPS to detect antibodies against linear and conformational epitopes. When being mixed with the tested samples, the luciferase-tagged antigen will form immunocomplex with antigen-specific antibodies, which can be captured by Protein A/G conjugated beads. The activity of luciferase-tagged antigen captured by the beads is correlated with specific antibody responses. LIPS assays have been widely used for antibody detection in autoimmune and infectious diseases (Burbelo et al., 2015; Shabani Azim et al., 2016). Studies showed that the detection sensitivity of LIPS is higher than ELISA for profiling human norovirus antibodies (Tin et al., 2017). In this study, we established a *Gaussia luciferase* (GLuc) immunoprecipitation assay for the serological detection of ASFV infection based on the immunogenic viral protein p30. Based on the ROC curve analysis, when the cutoff value of S/N (sample/negative) is set as >2.298 and ≤30.59, this LIPS assay can perfectly identify ASFV infection. In addition, no cross-reactivity to other common swine pathogens was observed in our hands. Therefore, this newly developed LIPS for the rapid and easy detection of ASFV antibodies could serve as another promising assay for ASF diagnosis and ASFV antibody surveillance.

## Materials and methods

### Cells, antibodies, and reagents

HEK-293T cells from American Type Culture Collection (ATCC; Manassas, VA, USA) were maintained in Dulbecco’s modified Eagle’s medium (DMEM; Hyclone, Logan, UT, USA) supplemented with 10% fetal bovine serum (FBS; Sigma-Aldrich, St Louis, MO, USA) and 1% penicillin-streptomycin (Thermo Fisher Scientific Waltham, MA). Coelenterazine h (Maokang Biotechnology, Shanghai, China) was used for the gaussia luciferase assay. Mouse monoclonal antibody against FLAG-tag (MBL, Nagoya, Japan), HRP-conjugated goat anti-mouse IgG (Sangon Biotech, Shanghai, China), and GAPDH monoclonal antibody (Bioworld Technology, Nanjing, China) were used in Western blot analysis. Alexa Fluor^®^ 488-conjugated goat anti-mouse IgG antibody (Jackson ImmunoResearch, West Grove, PA, USA) was used as the secondary antibody in the immunofluorescence assay.

### Pig serum samples

Thirty-seven ASFV-positive serum samples were provided by the African Swine Fever Regional Laboratory of China (Lanzhou), Lanzhou Veterinary Research Institute, Chinese Academy of Agricultural Sciences. Forty pig serum samples collected before the ASF outbreak in China were used and considered as negative samples. All serum samples were inactivated at 60°C for 30 min before use. Serum samples collected from pigs infected with a specific pathogen were used to evaluate the diagnostic specificity of the GLuc-p30-based LIPS assay, including 5 porcine reproductive and respiratory syndrome virus-positive (Li et al., 2022), 2 classical swine fever virus-positive provided by Dr. Qin Wang from China Institute of Veterinary Drugs Control, 5 Senecavirus A-positive serum provided by Dr. Zuzhang Wei from Guangxi University, and 3 porcine delta coronavirus-positive and 2 porcine epidemic diarrhea virus-positive provided by Dr. Yongning Zhang from China Agriculture University.

### Construction of plasmids expressing ASFV p30 and p54 fused with gaussia luciferase

The coding regions of *CP204L* (p30) and *E183L* (p54) from ASFV isolate Pig/HLJ/2018 (Genbank accession No. MK333180.1) were codon-optimized according to human codon usage, synthesized by Genscript (Nanjing, China), and cloned into pcDNA3.1(+)-P2A-eGFP vector, respectively. These two plasmids were digested with restriction enzymes *EcoR* V and *Xho* I, and the codon-optimized p30 and p54 genes were gel-purified and cloned into the pCAGGS-GLuc-FLAG vector using T4 DNA ligase (Vazyme Biochem, Nanjing, China). Finally, the plasmids for the expression of the fusion proteins, GLuc-p30 and GLuc-p54, were designated as pCAGGS-GLuc-p30 and pCAGGS-GLuc-p54 (**Figure 2A**). The correct insertion of p30 and p54 coding sequences was verified *via* DNA sequencing by Genscript (Nanjing, China).

### The expression of GLuc-p30 and GLuc-p54 in HEK-293T cells

The plasmids containing the codon-optimized ASFV p30 and p54 were transfected into HEK-293T cells to express the fusion proteins, GLuc-p30 and GLuc-p54. Briefly, HEK-293T cells at 80% confluency in the 6-well tissue culture plate were DNA transfected with 2µg plasmid per well using Lipofactemin 2000 (Thermo Fisher Scientific Waltham, MA) according to the manufacturer’s instructions. At 24, 48, and 72 hours post-transfection (hpt), culture supernatants were harvested, whereas cell lysates were harvested with IP lysis buffer (Biosharp, Hefei, China) supplemented with a protease inhibitor cocktail (Roche). Both culture supernatants and cell lysates were clarified by centrifugation at 12,000×g at 4°C for 10 min and then stored at -80°C. The expression of the fusion proteins was confirmed by gaussia luciferase assay, Western blot analysis, and immunofluorescence assay using a monoclonal antibody against the FLAG tag.

### Western blot analysis

Cell lysates and culture supernatants harvested at 48 hpt were mixed with 5X sample loading buffer (Beyotime Biotech, Shanghai, China) and boiled at 95°C for 5 min. Denatured proteins were separated by sodium dodecyl sulfate-polyacrylamide gel electrophoresis (SDS-PAGE) in 12% Tris-glycine gels and transferred to a nitrocellulose membrane. The membrane was blocked with 5% skim milk in phosphate-buffered saline (PBS) at 4°C overnight and then incubated with the anti-FLAG antibody diluted at 1:10000 at room temperature (RT) for 1 h. After five washes with PBS-0.05% Tween 20 (PBST), the membrane was incubated with a goat anti-mouse horseradish peroxidase (HRP)-conjugated secondary antibody diluted at 1:5000 at RT for 1 h. After five washes in PBST, the targeted proteins were visualized with the ECL chemiluminescence substrate (Vazyme Biotech, Nanjing, China) using a Tanon 5200 Multi Imaging system (Tanon, Shanghai, China). In addition, glyceraldehyde-3-phosphate dehydrogenase (GAPDH) was detected as a loading control using a rabbit polyclonal antibody (HuaBio, Hangzhou, China).

### Indirect immunofluorescence assay (IFA)

HEK-293T cells transfected with indicated plasmids in a six-well plate were fixed 48 hours post-transfection (hpt) with 4% (w/v) paraformaldehyde (PFA) in PBS for 20 min and then permeabilized with 0.1% (v/v) Triton X-100 for 10 min at RT. The cell monolayers were blocked with 1% bovine serum albumin (BSA) diluted in PBS for 30 min at RT. The mouse anti-FLAG antibody (MBL, Nagoya, Japan) diluted at 1:1000 was added to each well and incubated for 1 h at 37°C. After five washes with PBS, the cell monolayers were incubated with a goat anti-mouse IgG antibody conjugated with Alexa Fluor 488 at RT for 1 h. Following five washes with PBS, 4’6’-diamidino-2-phenylindole (DAPI) (Solarbio, Beijing, China) was added to stain the cell nucleus. The cell monolayers were observed under an inverted epifluorescence microscope IX73 (Olympus LS, Japan).

### Gaussia luciferase assay

The gaussia luciferase assay was performed with coelenterazine h substrate (Maokang Biochem, Shanghai, China) as described previously (Zhou et al., 2022). The substrate was prepared by diluting coelenterazine h stock to 20 μM with PBS supplemented with 5 mM NaCl, pH 7.2, and incubated at RT in the dark for 30 min. To measure the gaussia activity, mix 50 μL of 20 μM coelenterazine h with 20 μL sample in a white plate and acquire photon counts for 10 sec using a SuPerMax 3000FL (Flash Spectrum Biological Technology, Shanghai, China) plate reader.

### A GLuc-p30-based LIPS assay for ASFV antibody detection

**Figure 1:**
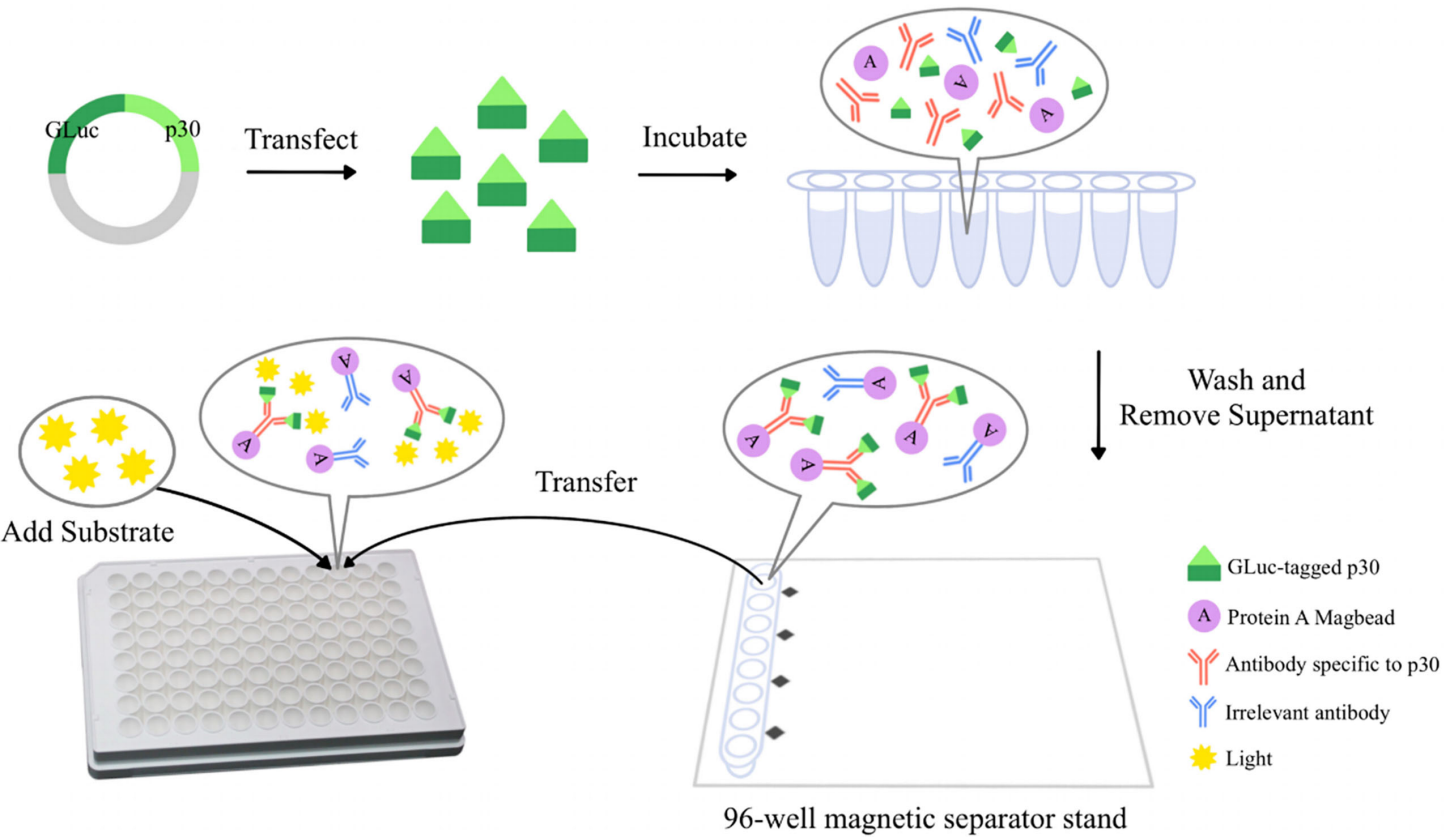
The schematic diagram of a GLuc immunoprecipitation assay for the detection of ASFV antibodies. The GLuc-p30 transiently expressed in HEK-293T cells is naturally secreted into the culture supernatant with the help of the GLuc secretory signal peptide. GLuc-p30 in culture supernatant is used directly for the GLuc immunoprecipitation assay without purification. Pig serum samples at appropriate dilution are mixed with GLuc-p30 and protein A Magbeads (Genscript, Nanjing, China) and then incubated in a rotator at 4 °C overnight. Magbeads are collected with a 96-well magnetic separator stand and washed extensively with PBST to remove the non-specific binding of GLuc-p30. Finally, the Magbeads are mixed with the substrate of GLuc to measure the luciferase activity of GLuc-p30 captured by p30-specific antibodies in the serum.

As shown in **Figure 1**, pig serum samples at an appropriate dilution were mixed with an indicated amount of GLuc-p30 protein and incubated on a rotator at 4°C overnight. To capture the antigen and antibody complex, added 5 μL of Protein A MagBeads (GenScript, Nanjing, China) to each reaction and incubated at RT for 1 h. After being washed five times with PBST, the magnetic beads were collected with a Magnetic Separator Stand 96-I (BEAVER biomedical, Suzhou, China) and transferred to a white 96-well plate for the gaussia luciferase assay.

### LIPS assay optimization

To get the optimum conditions of LIPS assay for ASFV antibody detection, one known ASFV-positive serum and one known ASFV-negative serum were used to check the signal-to-noise ratio that was calculated as P/N. The parameters tested with the highest P/N were chosen to set up the protocol for the LIPS assay. Initially, we optimized the input of GLuc-p30. Four different doses were tested, including 10^8^, 10^7^, 10^6^, and 10^5^ luminance units per reaction, and pig serum samples were diluted at 1:100. Next, the optimal dilution of serum samples for the LIPS assay was confirmed. A panel of serum dilutions was tested, including 1/50, 1/100, 1/200, 1/400, 1/800, 1/1600, and 1/3200, and the input of GLuc-p30 was 10^7^ luminance units per reaction.

### Determination of cutoff value for the LIPS assay

A total of 37 ASFV-positive and 40 ASFV-negative pig serum samples were utilized to determine the cutoff value for the LIPS assay. Serum samples were diluted at 1:100, and 10^7^ luminance units of GLuc-p30 were used per reaction. We determined the optimal cutoff value with the receiver operating characteristic (ROC) curve using the GraphPad Prism version 9.1.1 (GraphPad Software, San Diego, CA, USA). In addition, the cutoff value was also calculated as the mean+2SD.

### The diagnostic specificity of the LIPS assay

The diagnostic specificity of the GLuc-p30-based LIPS assay was evaluated with 17 pig serum samples collected from pigs infected with a specific pathogen as described in Pig serum samples.

### Indirect ELISA for ASFV antibody detection

A commercial indirect ELISA kit (Yisen-Bio, Beijing, China) using p30 as a diagnostic antigen was used to detect ASFV antibodies in serum samples according to the manufacturer’s instructions.

## Results

### The expression of GLuc-p30 and GLuc-p54 in HEK-293T cells

**Figure 2:**
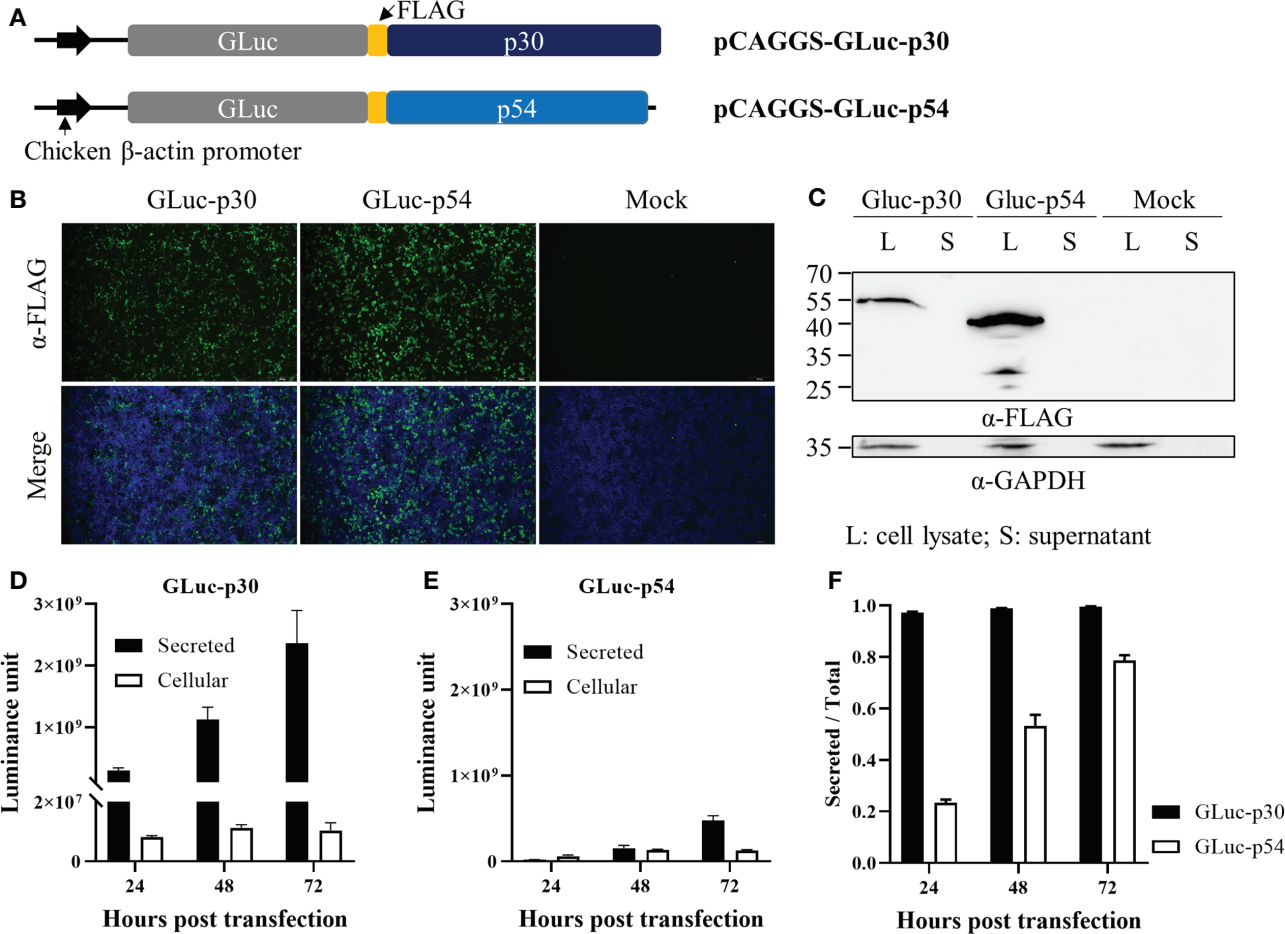
The expression of ASFV p30 and p54 as fusion proteins in HEK-293T cells. **(A)** A schematic representation of the plasmids expressing the fusion proteins, GLuc-p30 and GLuc-p54. HEK-293T cells were transfected with pCAGGS-GLuc-p30, pCAGGS-GLuc-p54, or pCAGGS vector. At 24, 48, and 72 hpt, culture supernatants were harvested, while cells were lysed with IP lysis buffer or fixed with 4% paraformaldehyde. The expression of GLuc-p30 and GLuc-p54 at 48 hpt was evaluated by IFA using an antibody against the FLAG tag **(B)** and western blot analysis using antibodies against the FLAG tag and GAPDH **(C)**. Luciferase assay was performed to measure GLuc-p30 **(D)** and GLuc-p54 **(E)** in cell lysates and supernatants at 24, 48, and 72 hpt. **(F)** The ratio of secreted GLuc-p30 and GLuc-p54. The data **(E, F)** represent the means ± SD (standard deviation) of triplicate.

To generate recombinant proteins for the LIPS assay, two eukaryotic plasmids were constructed for the expression of GLuc-p30 and GLuc-p54, as illustrated in **Figure 2A**. These plasmids were transfected into HEK-293T cells to prepare the fusion proteins. As shown in **Figure 2B**, the expression of the fusion proteins was confirmed by IFA using anti-FLAG mAb at 48 hpt. Western blot analysis also detected the fusion proteins in cell lysates but not in the culture supernatants (**Figure 2C**). Since GLuc is a naturally secreted protein, we also checked whether the fusion proteins could be secreted into the culture supernatant. The expression dynamics of the fusion proteins in culture supernatants and cell lysates at 24, 48, and 72 hpt were evaluated by gaussia luciferase assay. The expression levels of GLuc-p30 and GLuc-p54 increased gradually in culture supernatants from 24 hpt to 72 hpt, and GLuc-p30 exhibited much higher expression levels in culture supernatants (**Figures 2D, E**). As shown in **Figure 2F**, more than 97.3% of GLuc-p30 was secreted into culture supernatants at all time points, while the distribution of GLuc-p54 in culture supernatant increased gradually from 23.4% to 78.7%. Based on the expression level and distribution of the fusion proteins, the culture supernatant containing GLuc-p30 without purification was chosen as the antigen for the development of LIPS assay against ASFV.

### The establishment and optimization of the GLuc-p30-based LIPS assay for ASFV antibody detection

**Figure 3:**
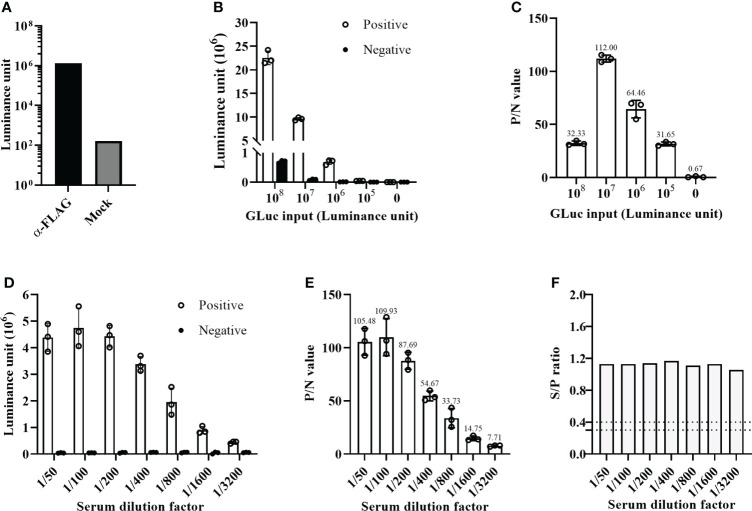
Optimization of the GLuc-p30-based LIPS assay. **(A)** The GLuc-p30-based LIPS recognized a mAb against the FLAG tag. **(B)** Optimization of GLuc-p30 input. Four different doses of GLuc-p30 were tested using an ASFV-positive serum and an ASFV-negative serum. The P/N ratios of the positive serum and the negative serum were calculated **(C)**. **(D)** Optimization of the serum dilution factor. Seven dilution factors were tested using 10^7^ LU per reaction. P/N ratios of the positive serum to the negative serum were calculated **(E)**. **(F)** The limit of detection of a p30-based indirect ELISA was evaluated with the same positive and negative serum. The S/P ratios were calculated for different serum dilutions. The data represent the means ± SD of triplicate.

Initially, we established the GLuc-p30-based LIPS assay using an anti-FLAG mAb, as shown in **Figure 1**. In comparison with the mock control, the anti-FLAG mAb was able to pull down GLuc-p30 efficiently as indicated by a ~10000-fold increase of luminance signal (**Figure 3A**).

To find the optimal detection conditions for the LIPS assay, we mainly optimized the input of GLuc-p30 and serum dilution factor. For this optimization, an ASFV-positive and an ASFV-negative pig serum were used, and the P/N ratio was used as the criteria to evaluate the signal-to-noise ratio. As shown in **Figure 3B**, with the decrease of GLuc-p30 input, the relative luciferase activities for both positive and negative sample detection were decreased. However, among the tested doses, 10^7^ luminance units of GLuc-p30 per reaction reached the highest P/N ratio of 112 (**Figure 3C**). Therefore, the optimal input of GLuc-p30 is 10^7^ luminance units.

Next, seven serum dilutions (1/50 ~ 1/3200) were tested to find the optimal serum dilution factor. In this experiment, 10^7^ luminance units of GLuc-p30 and the positive and negative serum samples mentioned above were used. As shown in **Figures 3D, E**, the optimal serum dilution factor is 1/100, which gives the highest P/N ratio of 109.93. In line with the results in **Figure 2B**, the luciferase activities of negative serum detections remained consistently low at all dilutions. In addition, we also compared the limit of detection between our LIPS assay and an ASFV p30-based indirect ELISA kit. Both methods were able to detect ASFV antibodies from 1/50 to 1/3200 dilutions of the positive serum. However, as the dilution factor increased, the gradual reduction of the P/N ratio was observed in our LIPS assay but not for the S/N ratio in the indirect ELISA (**Figures 3E, F**), suggesting that the GLuc-p30-based LIPS assay could be used for ASFV antibody detection and quantification.

### The cutoff value and diagnostic specificity of the GLuc-p30-based LIPS assay

**Figure 4:**
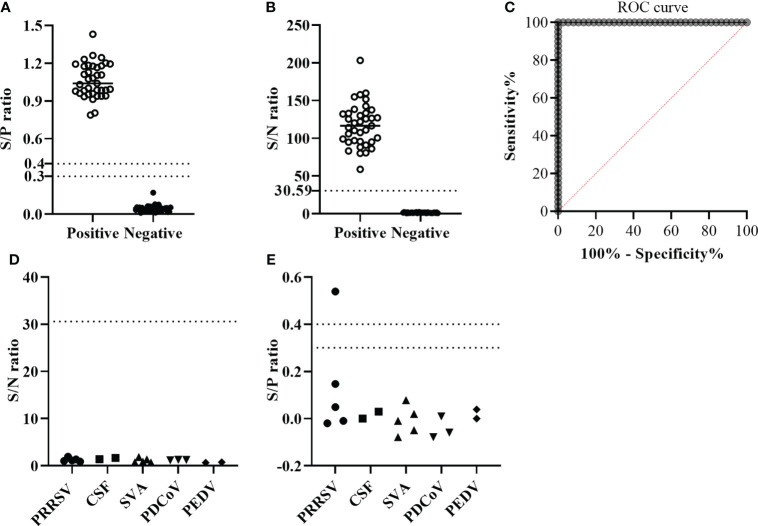
The performance of GLuc-p30-based LIPS with field serum samples. **(A)** S/P ratios were determined by a p30-based indirect ELISA to confirm the ASFV infection status of 37 ASFV-positive sera and 40 ASFV-negative sera. The cutoff value of the S/P ratio is between 0.3 and 0.4. **(B)** S/N ratios were obtained by the GLuc-p30-based LIPS assay of 37 ASFV-positive sera and 40 ASFV-negative sera. **(C)** The cutoff value of the LIPS assay was determined by ROC analysis. The diagnostic specificities of LIPS **(D)** and ELISA **(E)** were evaluated with 17 pig serum samples collected from pigs infected with specific swine pathogens, including PRRSV, CSFV, SVA, PDCoV, and PEDV.

A total of 77 pig serum samples were used to determine the cutoff value of the GLuc-p30-based LIPS assay, including 37 positive sera and 40 negative sera which were collected from ASFV-infected or non-infected pigs. Firstly, these serum samples have been verified by a commercial indirect ELISA for ASFV antibody detection (**Figure 4A**) according to the manufacturer’s instructions. As expected, this indirect ELISA was able to identify positive and negative samples. Based on the ROC curve generated with all of the S/N ratios, the cutoff values could be set >2.298 and ≤30.59 to reach 100% sensitivity and 100% specificity for this LIPS assay (**Figures 4B, C**).

Subsequently, to check the cross-activity against other common swine pathogens, we evaluated the diagnostic specificity of the GLuc-p30-based LIPS assay using 17 sera collected from pigs infected with one specific swine pathogen as described in the section of Materials and methods. The S/N ratios of all serum samples were below the cutoff value, suggesting that this LIPS assay exhibited excellent diagnostic specificity (**Figure 4D**). The ASFV infection status of these samples was also tested with a commercial indirect ELISA. Except for one PRRSV-positive serum, all samples were determined to be ASFV-negative (**Figure 4E**), indicating that this indirect ELISA exhibited cross-activity with other pathogens. This PRRSV-positive serum was confirmed as ASFV-negative by IFA (data not shown).

## Discussion

Due to the lack of an efficient ASF vaccine, early diagnosis and strict application of biosecurity measures to recognize infection, remove infected animals, and restrict animal movement are crucial for ASF control. Accurate and simple serological methods for antibody detection in body fluid or sera would be more informative and beneficial to curb the development of epidemics. Due to its advantages as compared to other serological assays such as ELISA or protein microarray, the LIPS assay has already been widely used for measuring humoral immune responses in autoimmune and infectious diseases (Burbelo et al., 2015). In this study, we established a GLuc immunoprecipitation assay for the serological detection of ASFV infection based on the immunogenic viral protein p30. The GLuc-tagged p30 antigen was transiently expressed in HEK-293T cells and efficiently secreted in the cell culture supernatant (**Figure 2**), which was directly used for LIPS assay development. The optimal GLuc-p30 input and serum dilution factor for this LIPS were 10^7^ LU and 1/100 dilution (**Figure 3**). This GLuc-p30-based LIPS can perfectly distinguish the known positive and negative pig sera, indicated by the 100% sensitivity and 100% specificity (**Figure 4C**). Moreover, in comparison with a well-validated indirect ELISA for ASFV antibody detection (**Figure 4E**), our LIPS assay demonstrated better diagnostic specificity (**Figure 4D**).

Various luciferase genes have been used as the reporter gene in LIPS assays, including Renilla *luciferase* (Berguido et al., 2021; Hachim et al., 2022), NanoLuciferase (Honda et al., 2021), and GLuc (Honda et al., 2021). GLuc from the marine copepod *Gaussia princeps* is naturally secreted from mammalian cells in an active form and can be released efficiently into the conditioned medium of mammalian cells (Tannous, 2009). In addition, GLuc is over 1,000 times more sensitive than the commonly used firefly luciferase and *Renilla* luciferase. Because of its small size, unique thermal stability, and genetically encoded secretion system, GLuc could further simplify the preparation of luciferase-tagged antigens. Studies on bioluminescence kinetics showed obvious positive cooperativity of *Gaussia* luciferase with coelenterazine at different coelenterazine concentrations (Larionova et al., 2018). In this study, we fused the GLuc gene with the codon-optimized ASFV CP204L gene or E183L gene to express GLuc-p30 and GLuc-p54 proteins. As expected, GLuc-p30 was efficiently expressed and secreted (more than 97%) into the culture supernatant. Nevertheless, the expression level of GLuc-p54 was much lower. In addition, the low secreted percentage of GLuc-p54 may be due to the transmembrane domain of p54. So we developed the LIPS assay only with GLuc-p30 protein. Since p54 is also an immunogenic ASFV protein and has been used for ELISA development, we will continue to optimize the expression and secreted level of GLuc-p54 for LIPS assay development in our future study. Compared to the luciferase-tagged antigens utilizing other luciferase genes (non-secreted), the preparation of GLuc-tagged antigens can be easier and less time-consuming.

Recently, another research group established an MP-LIPS assay for the detection of ASFV antibodies using a similar strategy (Liu et al., 2021). They also chose ASFV p30 as the target antigen and purified firefly luciferase tagged p30 from *Escherichia coli* for LIPS assay, which is more complicated and time-consuming than our preparation of GLuc-p30 in the eukaryotic expression system. The optimal P/N ratio (about 100) of this LIPS is very similar to our results (**Figure 3**). However, the sensitivity and specificity of this LIPS assay are much lower than those of our GLuc-p30-based LIPS, although less serum samples were tested in this study. ASFV p30 is usually expressed in *Escherichia coli* as inclusion bodies and can be purified when denatured. However, only a small part of denatured protein can be refolded to its native confirmation. Therefore, we believe that the native conformation of the luciferase-tagged p30 used for LIPS is essential for the recognition of various antibodies against this antigen in samples. Since this MP-LIPS is semi-automated and can be finished in 30 min, we will further optimize our GLuc-p30-based LIPS assay with a fully automated system to decrease the detection time in the future.

The performance of the GLuc-p30-based LIPS assay was compared with a commercial indirect ELISA kit using p30 as the diagnostic antigen. Using a panel of known positive and negative serum samples confirmed with this ELISA kit, our LIPS assay can correctly identify samples with ASFV antibodies. Our LIPS exhibited no cross-reactivity to other common swine pathogens, while the ELISA recognized a sample with PRRSV infection as ASFV-positive. Consequently, the diagnostic sensitivity of this LIPS is comparable to that of this commercial indirect ELISA, while the specificity of LIPS is even superior to the ELISA. In addition to serum, oral fluid is another sample used for the diagnosis of infectious diseases in the population because its collection requires little labor and is stress-free for animals. Since ASFV antibodies could be detected in oral fluids, oral fluids could be a suitable diagnostic specimen for ASFV surveillance (Olsen et al., 2013). We will test whether our LIPS assay can be used with pig oral fluids.

In conclusion, we have established a GLuc-p30-based LIPS assay that is a cheap, easy-to-implement and highly adaptable method for large-scale screening of ASFV antibodies. Because of its similar diagnostic sensitivity and superior diagnostic specificity to the commercial indirect ELISA, this LIPS could be a potential method for ASFV diagnosis in laboratories and farms.

## Data availability statement

The datasets presented in this study can be found in online repositories. The names of the repository/repositories and accession number(s) can be found in the article/supplementary material.

## Author contributions

YL: conceptualization, supervision, founding acquisition. YL and JD wrote the original draft. JD, JY, and DJ: investigation. JD and DJ: methodology, formal analysis, and data curation. JD, JY, and YL reviewed and edited the manuscript. JY contributed to the resources. All authors contributed to the article and approved the submitted version.

## Funding

This project was supported by the Key Research and Development Plan (Modern Agriculture) of Jiangsu Province (Grant No. BE2020398), Jiangsu Co-innovation Center for Prevention and Control of Important Animal Infectious Diseases and Zoonoses, and the Priority Academic Program Development of Jiangsu Higher Education Institutions (PAPD). JD is supported by the Postgraduate Research & Practice Innovation Program of Jiangsu Province (Grant No. KYCX22_3551). YL is supported by the Scientific Research Foundation of Yangzhou University and the “LvYangJinfeng Program” of Yangzhou City.

## Acknowledgments

We thank Dr. Adolfo García-Sastre from Icahn School of Medicine at Mount Sinai, New York for kindly sharing the pCAGGS vector.

## Conflict of interest

The authors declare that the research was conducted in the absence of any commercial or financial relationships that could be construed as a potential conflict of interest.

## Publisher’s note

All claims expressed in this article are solely those of the authors and do not necessarily represent those of their affiliated organizations, or those of the publisher, the editors and the reviewers. Any product that may be evaluated in this article, or claim that may be made by its manufacturer, is not guaranteed or endorsed by the publisher.
